# Effect of tobacco smoking on the risk of developing community acquired pneumonia: A systematic review and meta-analysis

**DOI:** 10.1371/journal.pone.0220204

**Published:** 2019-07-18

**Authors:** Vadsala Baskaran, Rachael L. Murray, Abby Hunter, Wei Shen Lim, Tricia M. McKeever

**Affiliations:** 1 Department of Respiratory Medicine, Nottingham University Hospitals NHS Trust, Nottingham, United Kingdom; 2 Division of Epidemiology and Public Health, University of Nottingham, Nottingham, United Kingdom; Instituto Butantan, BRAZIL

## Abstract

**Aim:**

To summarise and quantify the effect of tobacco smoking on the risk of developing community acquired pneumonia (CAP) in adults.

**Methods:**

We systematically searched MEDLINE, Embase, CINAHL, PsychINFO and Web of Science, from inception to October 2017, to identify case-control and cohort studies and reported in accordance with the Preferred Reporting Items for Systematic Reviews and Meta-Analysis (PRISMA) checklist. The review protocol was registered with the PROSPERO database (CRD42018093943). Study quality was assessed by the Newcastle-Ottawa Scale. Pooled odds ratios (ORs) or hazard ratios (HRs) were estimated using a random-effects model.

**Results:**

Of 647 studies identified, 27 studies were included (n = 460,592 participants) in the systematic review. Most of the included studies were of moderate quality with a median score of six (IQR 6–7). Meta-analysis showed that current smokers (pooled OR 2.17, 95% CI 1.70–2.76, n = 13 studies; pooled HR 1.52, 95% CI 1.13–2.04, n = 7 studies) and ex-smokers (pooled OR 1.49, 95% CI 1.26–1.75, n = 8 studies; pooled HR 1.18, 95% CI 0.91–1.52, n = 6 studies) were more likely to develop CAP compared to never smokers. Although the association between passive smoking and risk of CAP in adults of all ages was not statistically significant (pooled OR 1.13, 95% CI 0.94–1.36, n = 5 studies), passive smoking in adults aged ≥65 years was associated with a 64% increased risk of CAP (pooled OR 1.64; 95% CI 1.17–2.30, n = 2 studies). Dose-response analyses of data from five studies revealed a significant trend; current smokers who smoked higher amount of tobacco had a higher risk of CAP.

**Conclusion:**

Tobacco smoke exposure is significantly associated with the development of CAP in current smokers and ex-smokers. Adults aged > 65 years who are passive smokers are also at higher risk of CAP. For current smokers, a significant dose-response relationship is evident.

## Introduction

Community acquired pneumonia (CAP) is a common communicable disease with an estimated annual age-adjusted incidence of 465–649 patients hospitalised with CAP per 100,000 population in the United States. [1,2] In 2016, lower respiratory tract infections (LRTIs) including CAP were reported as the ‘most deadly communicable disease’ worldwide, causing three million deaths and were the second commonest reason for years of life lost after ischaemic heart disease.[3,4]

Tobacco smoking is a major cause of morbidity and mortality in high income countries and is an important risk factor for CAP.[5] Tobacco smoking impairs mucociliary clearance by causing an increase in mucous production and number of abnormal cilia alongside reduction of ciliary beat frequency.[6] Piatti *et al*. found that tobacco smoking modifies buccal epithelial surfaces which causes increased pneumococcal adherence compared to never smokers.[7] Greater bacterial adherence may lead to greater oropharyngeal colonisation and hence a greater risk of developing CAP. Exposure to low levels of tobacco smoking or passive smoking has also been shown to be associated with modification in the lung cell biology similar to that seen in current smokers. [8,9]

A recent systematic review of risk factors for CAP in adults published by Almirall *et al*. found that tobacco smoking was a significant risk factor for CAP compared to never smokers, however the strength of association was not quantified.[10] Passive smoking has been shown to increase the risk of lower respiratory tract infections (LRTIs) in children whose parents smoke[11], yet there has been no systematic review to summarise the risk for developing CAP in adults.

The aim of this systematic review and meta-analyses was to summarise the available evidence regarding the effect of tobacco smoking and passive smoke exposure on the risk of developing CAP in adults, to determine the strength of the association and to examine whether there is a ‘dose-response’ association between amount of tobacco smoked and the risk of developing CAP.

## Methods

This systematic review was conducted using a predefined protocol which was registered with PROSPERO database (CRD42018093943) and reported in accordance with the Preferred Reporting Items for Systematic Reviews and Meta-Analysis (PRISMA) statement.

### Search strategy and study selection

The search strategy was designed to find published studies. Comprehensive searches of the following biomedical electronic databases were conducted: MEDLINE, Embase, CINAHL, PsycINFO and Web of Science from the commencement of these databases to October 2017. The search strategy included subject headings and keywords related to “community acquired pneumonia” and “smoking”. Search keywords were determined from the Cochrane review group terms for tobacco.[12] Details of the search strategy for each database are found in S1 File. The reference list of all included studies were screened for inclusion.

This review included observational studies; prospective and retrospective cohort studies and case-control studies. Cross-sectional studies were excluded. Studies published in all languages were considered and no date restrictions were placed on searches. Studies comprising adults aged 15 years and above with either a clinical or radiology-confirmed diagnosis of CAP were included. Studies comprising patients with hospital acquired pneumonia, aspiration pneumonia, active pulmonary TB and post-obstructive pneumonia secondary to thoracic malignancy were excluded.

Two authors (VB, AH, RM or TM) independently screened titles and abstracts using Covidence software[13] and subsequently reviewed full-texts of retrieved studies for eligibility. Disagreement was resolved by discussion and consensus, involving a third reviewer (TM or WSL) where necessary.

### Data extraction and assessment of methodological quality

Two authors (VB, RM or TM) independently extracted all data for studies in English whilst data from non-English studies were extracted by a single reviewer (TL, LB, MOB or KN) who was literate in that particular language using a standardised form. Any disagreements that arose between reviewers were resolved through discussion, or with a third reviewer (TM) when required. Information on study population, study design, exposure of interest (tobacco smoking) including different exposure categories (e.g. never, ever, ex-, current, ‘not current’ and passive smokers), outcome (CAP) and the adjusted/ unadjusted effect size (either odds ratios (ORs) or hazard ratios (HRs)) were collected.

Methodological quality was assessed using the Newcastle-Ottawa Quality Assessment Scale[14] for either cohort or case-control studies depending on individual study design. This scale is based on three broad categories; (1) selection of the study sample (four points), (2) comparability of the sample groups (two points) and (3) ascertainment of exposure/ outcome (three points). Thus, studies were scored out of a total of nine points. Scores were chosen *a priori* to indicate different levels of methodological quality (0–3: low quality, 4–6: moderate quality, 7–9: high quality)

### Data synthesis

We reviewed the extracted results to assess if adequate similarity existed for study outcomes to conduct a random-effects meta-analysis using Stata/ SE 15.1 (StataCorp. 2017). Meta-analysis was performed using 26 studies comparing current (selecting the highest amount of tobacco smoked for current smokers where there was more than one category), ever, ex- and passive versus never smokers in addition to comparing current versus ‘not current’ smokers. We assessed publication bias visually using a funnel plot for the association between current smoking and the risk of CAP given that there were more than 10 studies included in this meta-analysis. We summarised studies with pooled ORs and HRs separately with 95% confidence intervals. Measures of effect adjusted for confounders (age and sex were *a priori* confounders) were used in preference to crude measures of effect. The I^2^ statistic was used to assist with assessment of heterogeneity between studies. We performed sensitivity analysis excluding studies with only specific medical conditions (we use the term ‘selected clinical populations’ in the rest of this paper) so that the data would be representative of the general population. In order to explore ascertainment bias, we compared the effect size in primary care versus secondary care settings. We plotted the log ratio for each category within a study (assuming a linear relationship) and estimated the dose-response regression coefficient to determine the dose-response association between the dose of current smoking and the risk of developing CAP.

## Results

**Fig 1:**
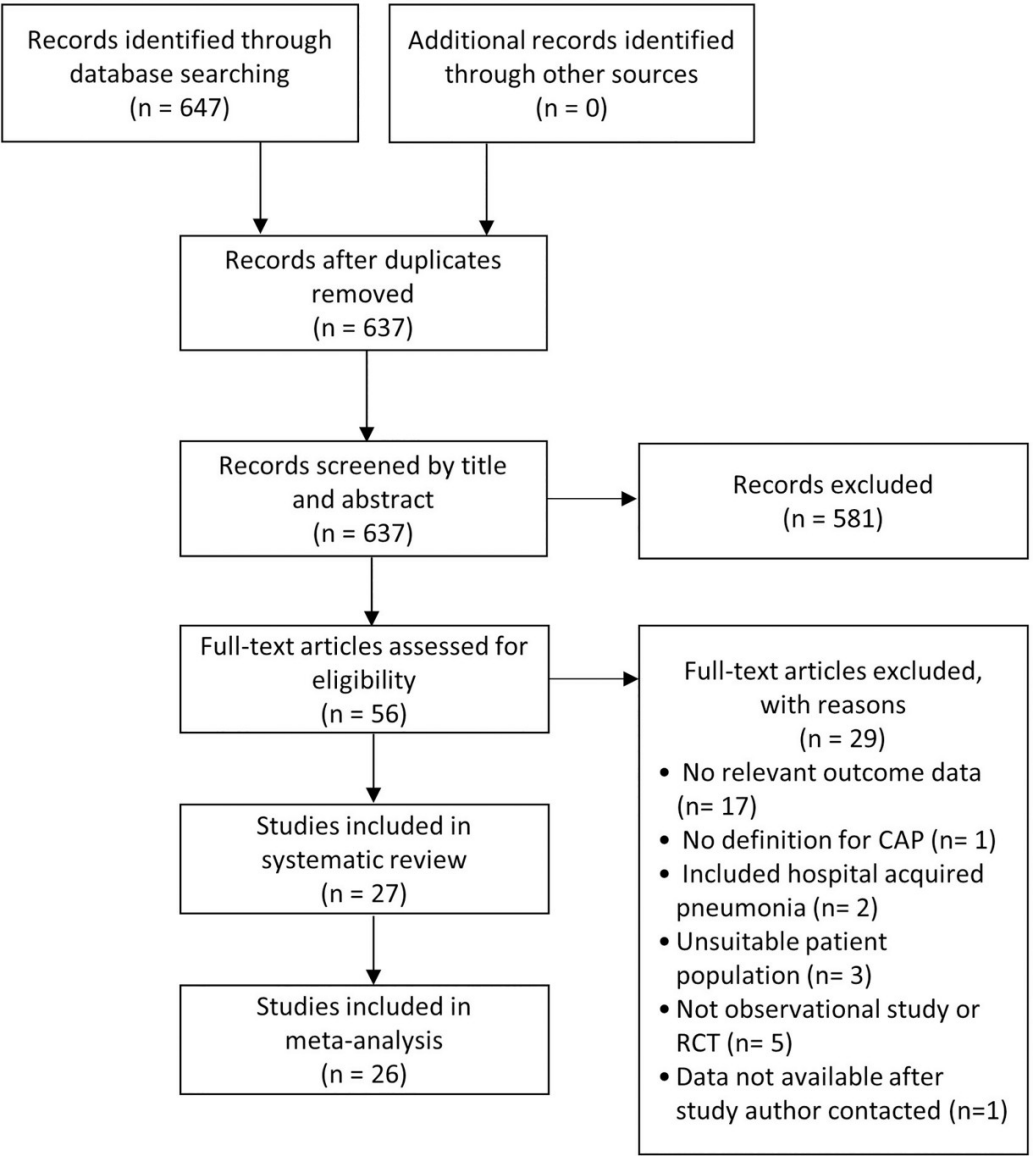
PRISMA flow diagram for study selection.

The search strategy initially identified 647 studies, from which 56 full-text articles (including five non-English studies; French, Chinese, German and Spanish) were reviewed (Fig 1). Twenty five English studies and two non-English studies were included in the systematic review (n = 460,592 participants) (S1 Table). The most common reason for exclusion was lack of documented relevant outcome data (n = 17/56).

### Characteristics of included studies

Of 27 included studies, there were 13 cohort studies and 14 case-control studies. All except two studies included both genders; two studies only included men.[15,16] Five studies had selected clinical populations including patients with human immunodeficiency virus (HIV)[17], selected HIV-related medical conditions[16], minor thoracic injury[18] and chronic obstructive pulmonary disease (COPD)[19,20]. One study reported an outbreak of Legionnaires’ disease and included participants who visited an aquarium.[21] Six studies were conducted in primary care, nine in hospitals, four in mixed settings, six in community settings and two not specified (S1 Table). The definition of CAP was based on radiological confirmation in 16 studies, diagnostic coding in five studies (four studies used ICD-9 codes and one study used Clinical Practice Research Datalink read codes) and clinical criteria in five studies; one study did not report the way CAP was defined.

### Risk of bias

Most of the studies (n = 16 studies) included were of moderate quality with a median quality score of six (IQR 6–7) (S2 Table). Using the a priori methodological quality scores, we judged ten studies to be of high quality and one study of low quality. Twenty-four studies (88.9%) had clear definitions of CAP either using independent blind assessment of chest radiographs, medical records or record linkage (e.g. ICD codes) and 23 studies (85.2%) adjusted for confounders. Thirteen (92.9%) out of all case-control studies scored low as a result of lack of non-response rate reporting and poor smoking status ascertainment (obtained information from interview that was not blinded to case/ control status or were self-reported). In contrast, over half of the cohort studies (n = 8 studies, 61.5%) scored well for smoking status ascertainment; either from a secure medical record or structured interview with the participant. The quality for some of the cohort studies dropped due to a combination of lacking a truly representative exposed cohort (n = 8 studies, 61.5%), not demonstrating that CAP was not present at the start of the study (n = 8 studies, 61.5%) and not having a statement about loss to follow-up (n = 9 studies, 69.2%).

### Smoking status

The most commonly used definitions of smoking status are detailed in Table 1. Most studies state the smoking categories without providing detailed definition for respective categories; we have used the definition for each category from the Glossary detailed in the Adult Tobacco Use Information by the Centers for Disease Control and Prevention (Table 1).[22]

**Table 1:** Smoking categories used in included studies.

Smoking categories	Definition[22]	Synonyms used in included studies	Number of studies
Never	Never smoked or lifetime smoking history of <100 cigarettes	Non	27
Ever	Lifetime smoking history of ≥100 cigarettes	‘Current/ past’ ‘Current/ ex’	4
Ex	Lifetime smoking history of ≥100 cigarettes and stopped smoking at the time of the study	Former, past	15
Current	Lifetime smoking history of ≥100 cigarettes and currently smokes at the time of the study	Active	18
Passive	Never smokers who are exposed to environmental cigarette smoke	Exposure to second-hand smoke	6

Four studies quantified tobacco smoking by documenting pack-years[23,24] and reporting qualitative descriptions from light through to heavy smoking.[16,25] ‘Not current’ smoking category which could include never, ever and ex-smokers was used in two studies. The proportion of current smokers with CAP was higher in secondary care (31–79%) compared to primary care (21–27.3%).

### Meta-analyses

**Fig 2:**
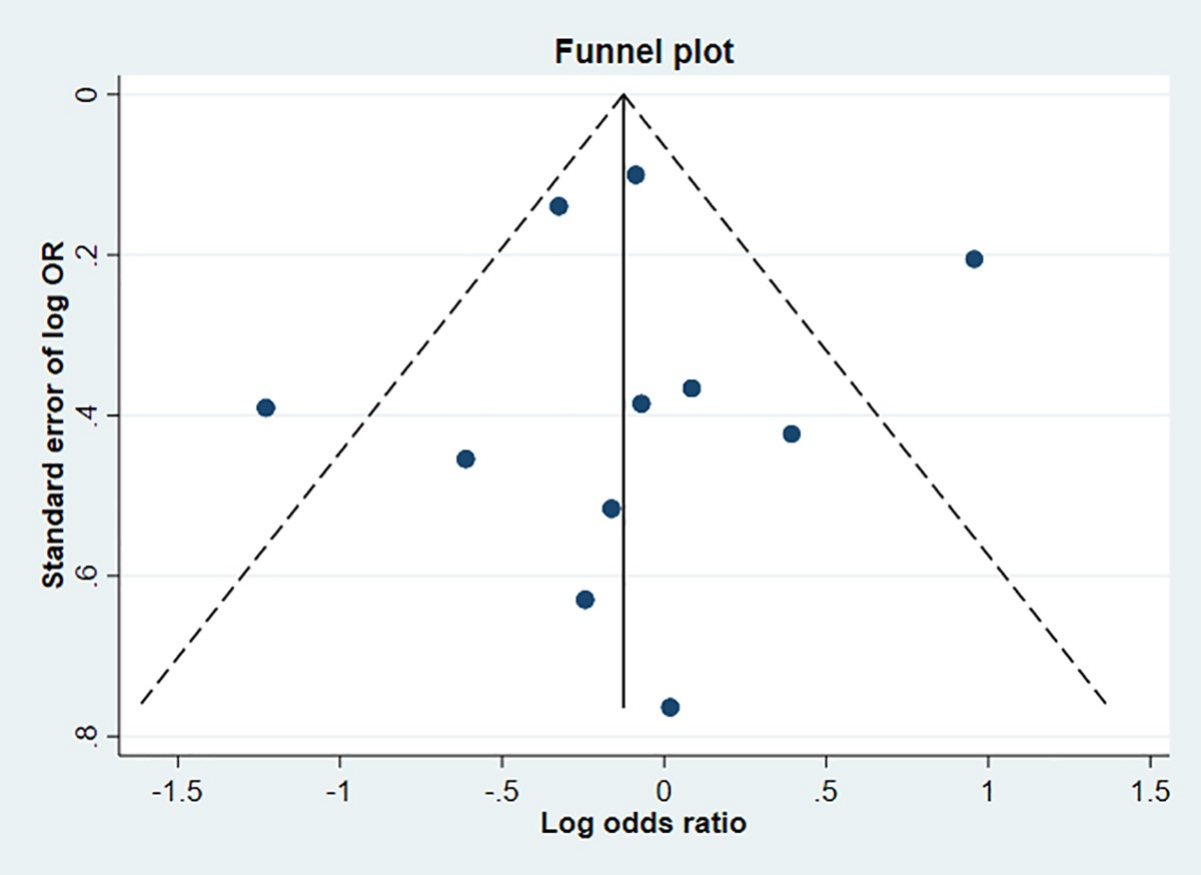
Meta-analysis of risk of community acquired pneumonia in current smokers relative to never smokers (Odds Ratio). * Study by Baik *et al*. had relevant data subdivided by gender, therefore data from this study were included as two separate entities (i.e. men and women). Grey box = effect estimates from single studies. Diamond = pooled result with confidence interval. Vertical line at ‘1’ on the x-axis is the line of no effect. Weight (in %) = influence an individual study had on the pooled result.

**Fig 3:**
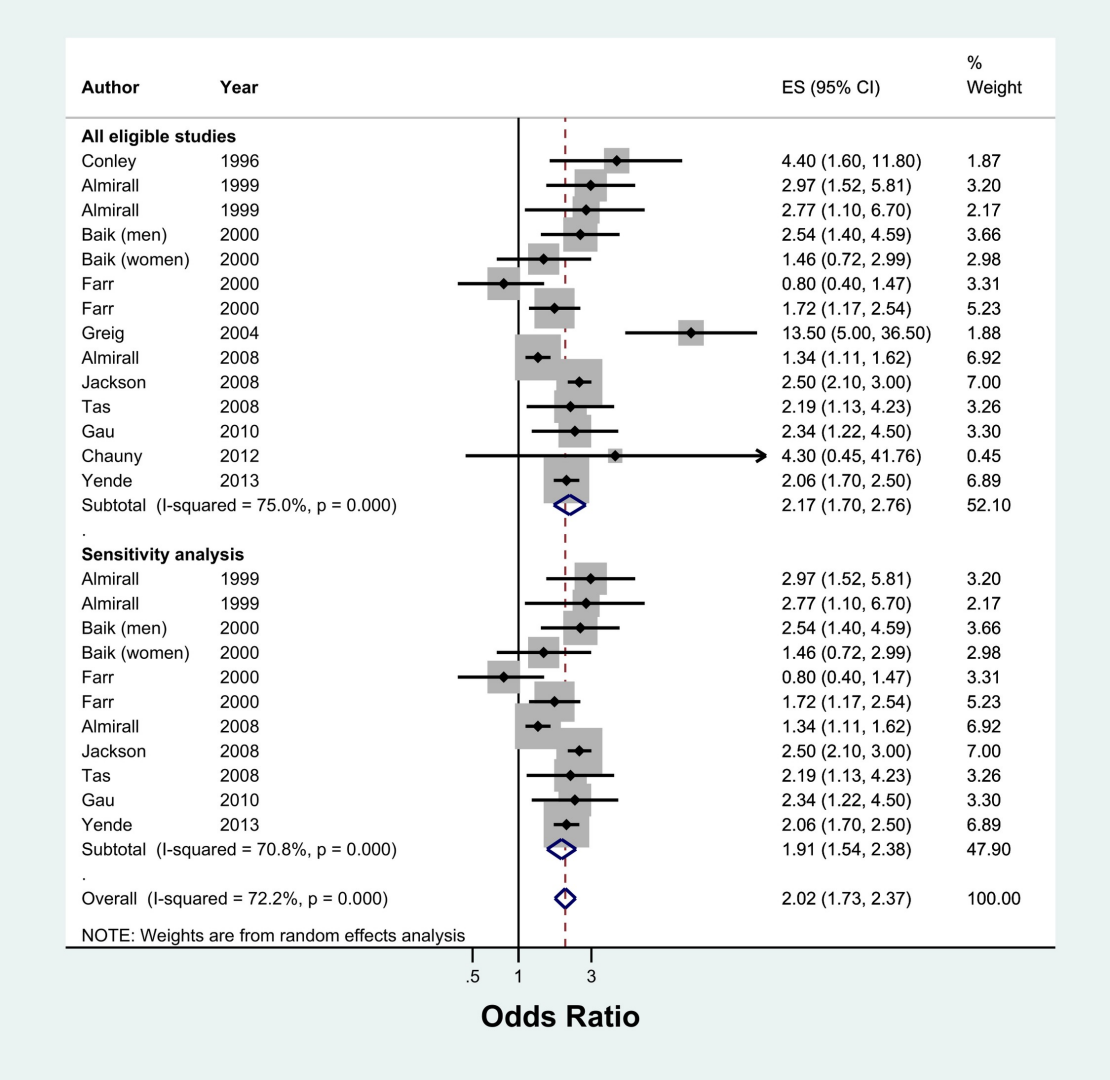
Funnel plot for the association between current smoking and the risk of developing CAP.

**Fig 4:**
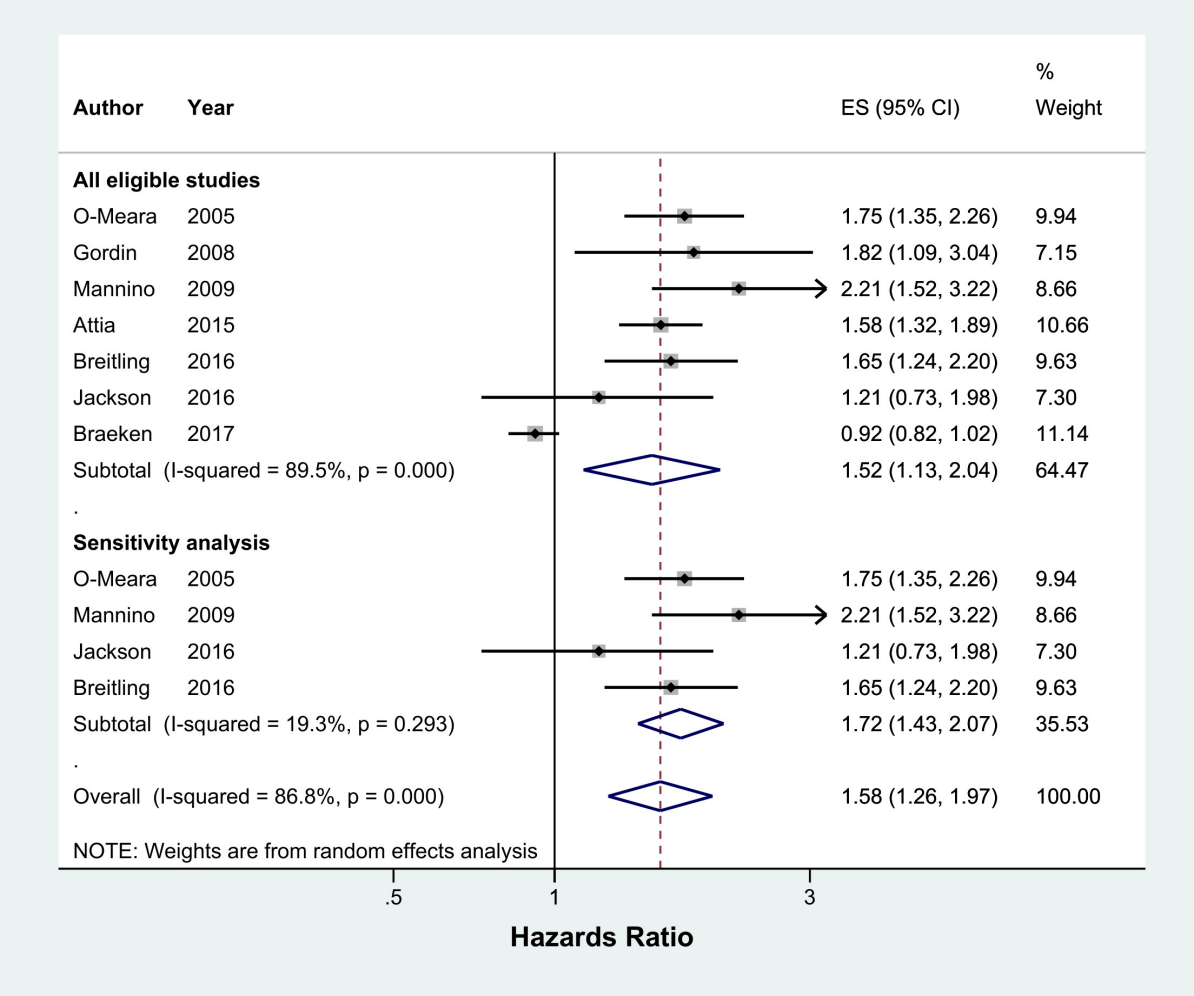
Meta-analysis of incidence of community acquired pneumonia in current smokers relative to never smokers (Hazards Ratio). Grey box = effect estimates from single studies. Diamond = pooled result with confidence interval. Vertical line at ‘1’ on the x-axis is the line of no effect. Weight (in %) = influence an individual study had on the pooled result.

**Fig 5:**
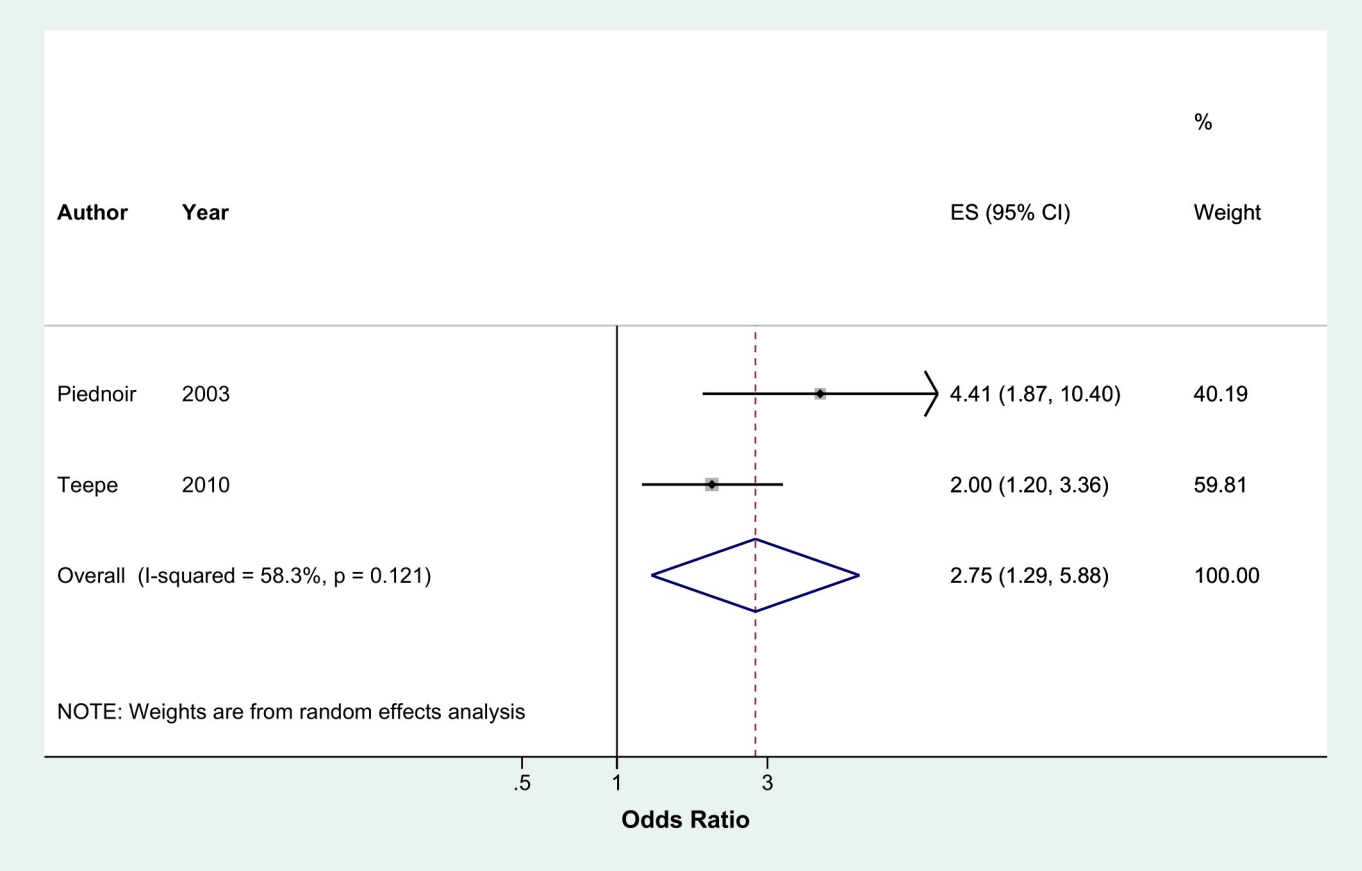
Meta-analysis of risk of community acquired pneumonia in current smokers relative to 'not current' smokers (Odds Ratio). Grey box = effect estimates from single studies. Diamond = pooled result with confidence interval. Vertical line at ‘1’ on the x-axis is the line of no effect. Weight (in %) = influence an individual study had on the pooled result.

**Fig 6:**
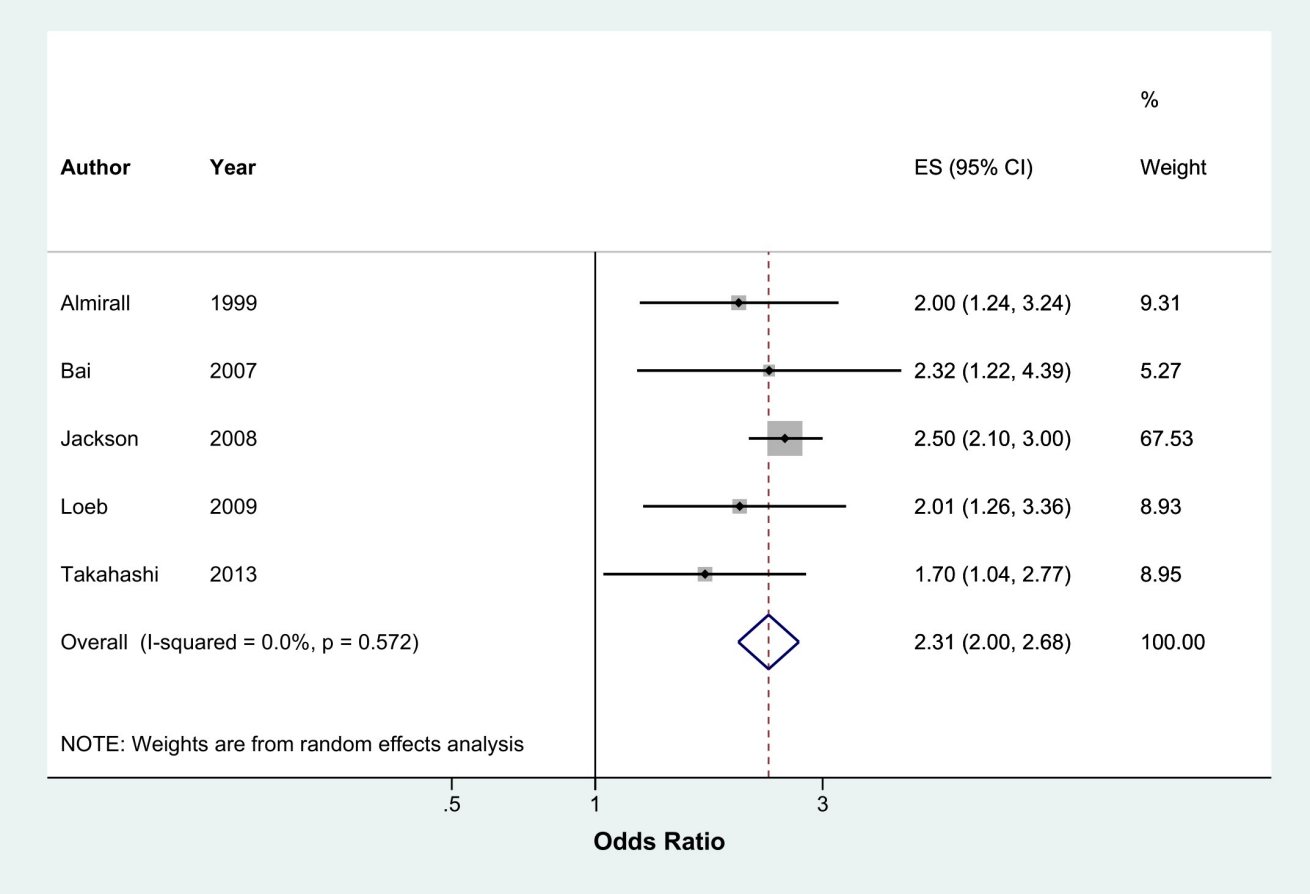
Meta-analysis of risk of community acquired pneumonia in current smokers relative to 'ever' smokers (Odds Ratio). Grey box = effect estimates from single studies. Diamond = pooled result with confidence interval. Vertical line at ‘1’ on the x-axis is the line of no effect. Weight (in %) = influence an individual study had on the pooled result.

Meta-analysis of 13 studies showed that current smokers were more than twice at risk of developing CAP than never smokers (pooled OR 2.17, 95% CI 1.70–2.76, I^2^ = 75%) (Fig 2). Sensitivity analysis excluding studies which were not representative of the general population (two studies with selected clinical populations[16,18] and one study which recruited participants who visited an aquarium[21]) found a marginally lower effect (pooled OR 1.91, 95% CI 1.54–2.38, I^2^ = 70.8%, n = 10 studies) (Fig 2). There was no evidence of publication bias identified from the funnel plot for the association between current smoking and the risk of CAP (Fig 3). Studies that reported hazards ratios found that current smokers were 53% more likely to develop CAP than never smokers (pooled HR 1.52, 95% CI 1.13–2.04, I^2^ = 89.5%, n = 7 studies) (Fig 4). The risk increased slightly when three studies with selected clinical populations were removed (pooled HR 1.72, 95% CI 1.43–2.07, I^2^ = 19.3%) (Fig 4). Compared to ‘not current’ smokers, meta-analysis of two studies revealed that current smokers were almost three times at risk of developing CAP (pooled OR 2.75, 95% CI 1.29–5.88, I^2^ = 58.3%, n = 2 studies) (Fig 5). There was only one study which compared current smokers to ‘not current’ smokers and reported hazards ratio (HR 1.31, 95% CI 1.17–1.46), hence this study was not included in the meta-analysis. [26] Meta-analysis of four studies showed that ever smokers were more than twice at risk of developing CAP than never smokers (pooled OR 2.31, 95% CI 1.99–2.69, I^2^ = 0%, n = 4 studies) (Fig 6).

**Fig 7:**
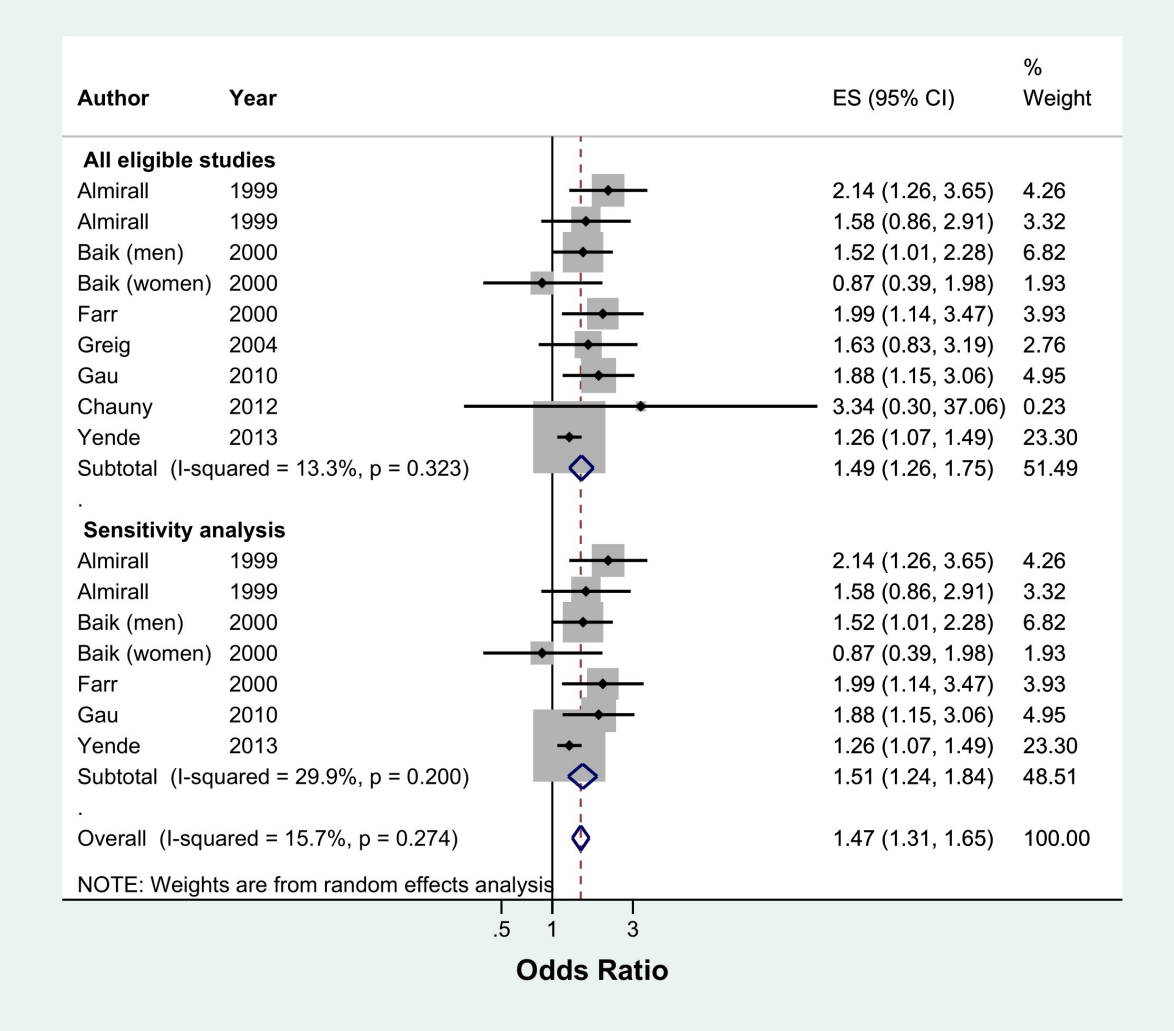
Meta-analysis of risk of community acquired pneumonia in ex-smokers relative to never smokers (Odds Ratio). Grey box = effect estimates from single studies. Diamond = pooled result with confidence interval. Vertical line at ‘1’ on the x-axis is the line of no effect. Weight (in %) = influence an individual study had on the pooled result.

**Fig 8:**
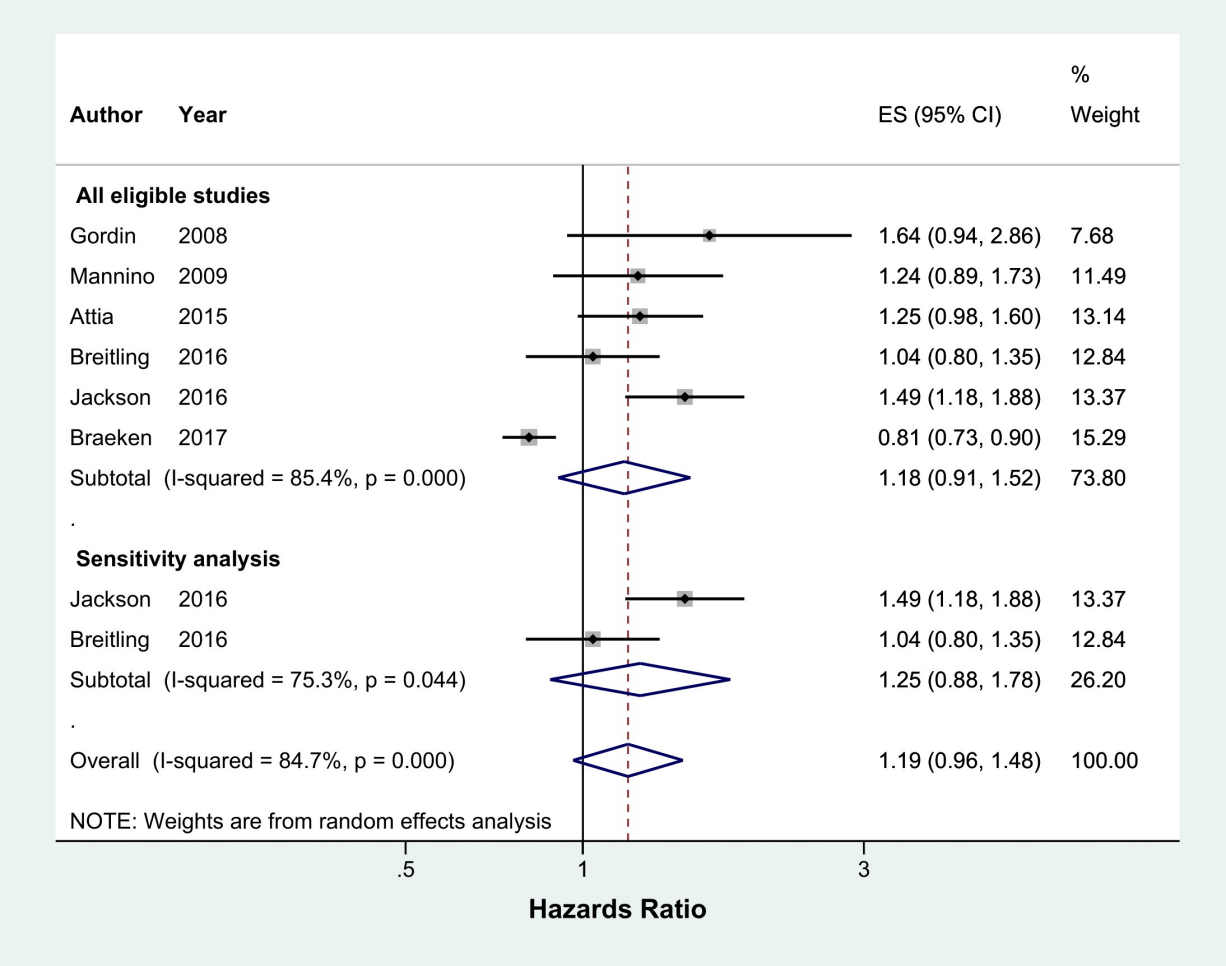
Meta-analysis of incidence of community acquired pneumonia in ex-smokers relative to never smokers (Hazards Ratio). Grey box = effect estimates from single studies. Diamond = pooled result with confidence interval. Vertical line at ‘1’ on the x-axis is the line of no effect. Weight (in %) = influence an individual study had on the pooled result.

Ex-smokers were 49% more likely to develop CAP than never smokers (pooled OR 1.49, 95% CI 1.26–1.75, I^2^ = 13.3%, n = 8 studies) (Fig 7). Sensitivity analysis excluding two studies which were not representative of the general population found a similar result (pooled OR 1.51, 95% CI 1.24–1.84, I^2^ = 29.9%, n = 6 studies) (Fig 7). Studies reporting hazard ratios had a high level of heterogeneity and did not find a significant effect between ex-smokers and the risk of developing CAP (All eligible studies: pooled HR 1.18, 95% CI 0.91–1.52, I^2^ = 85.4%, n = 6 studies and sensitivity analysis: pooled HR 1.25, 95% CI 0.88–1.78, I^2^ = 75.3%, n = 2 studies) (Fig 8).

For studies conducted only within a primary care setting, the effect of current smoking on the risk of developing CAP was not statistically significant (pooled OR 1.14, 95% CI 0.72–1.82, I^2^ = 55.1%, n = 2 studies and pooled HR 1.21, 95% CI 0.68–2.15, I^2^ = 92.8%, n = 2 studies) whereas for studies conducted in a secondary care setting, current smoking was significantly associated with CAP compared to never smokers (pooled OR 1.95, 95% CI 1.45–2.62, I^2^ = 0.0%, n = 4 studies and HR 1.58, 95% CI 1.32–1.89, n = 1 study).

Passive smoking was associated with a 13% increased risk of developing CAP compared to those who were not exposed to passive smoking, however this result was not statistically significant (pooled OR 1.13, 95% CI 0.94–1.36, I^2^ = 26.8%, n = 5 studies). In a sensitivity analysis of those ≥ 65 years old, passive smoking was associated with 64% increased risk of CAP (pooled OR 1.64; 95% CI 1.17–2.30, I^2^ = 0%, n = 2 studies).

### Dose-response trend: Narrative review

Five studies had information on the dose-response association between amount of smoking and the risk of developing CAP. We performed dose-response analyses using the data arising from these five studies (Table 2) although a meta-analysis was not possible due to variations in the method of quantification of smoking exposure across studies. Significant dose-response relationships were found in all studies; there were two linear[23,27] and three non-linear[16,25,28] associations with the effect in one study being mainly driven by results from the highest two categories (Table 2).[25]

**Table 2:** Dose-response relationship between amount of smoking and risk of developing CAP. Trend (OR) of 1.xy means xy% increase of risk of CAP per increase in category documented in the ‘Quantification of smoking exposure’ column.

Study	Smoking status	Quantification of smoking exposure	Trend (OR)	p value	Association
Almirall 1999	Current & ex-smokers	Pack-years • 0 • 5–16.4 • 16.5–38 • >38	1.37, 95% CI 1.17–1.61	<0.001	Linear
Almirall 2008	Current & ex-smokers	Packs of cigarettes smoked daily x 365 x years smoked^**$**^ • 0 • 1–150 • 151–300 • >300	1.27, 95% CI 1.17–1.38	<0.001	Linear
Almirall 1999	Current smokers	Cigarettes smoked daily • 0 • 1–9 • 10–20 • >20	1.30, 95% CI 1.02–1.67	0.037	Non-linear
Farr 2000	Current smokers	Cigarettes smoked daily x years smoked • 0 • 1–225 • **226–578** • **579+**	1.52, 95% CI 1.30–1.78	<0.001	Non-linear; effect mainly in the highest two categories of smoking (in **bold**)
Conley 1996	Current smokers	Packets of cigarettes daily • ≤ half (‘light’) • half to <2 (‘moderate’) • >2 (‘heavy’)	1.51, 95% CI 1.10–2.08	0.011	Non-linear

^**$**^This formula was clarified directly by personal correspondence with study author.

## Discussion

To our knowledge, this is the first study to quantify the effect of tobacco smoking on the risk of developing CAP through a meta-analysis. Our study revealed robust evidence that current and ex-smokers are significantly at higher risk of developing CAP whilst passive tobacco smoke exposure had a significant effect only in those aged ≥ 65. The strongest associations were evident in studies conducted in secondary care. In addition, a dose-response trend with higher risk of CAP amongst current smokers who smoke higher amounts of tobacco was noted.

Current smoking has been associated with a wide spectrum of infectious diseases including bacterial pathogens (*Streptococcus pneumoniae*, *Haemophilus influenzae*, *Neisseria meningitidis*, *Staphylococcus aureus*, *Legionella pneumophila*, *Mycobacterium tuberculosis*) and viral pathogens (influenza, rhinovirus, HIV).[29–34] Whether smoking increases the risk of infection from different respiratory pathogens by the same degree could not been fully examined in this systematic review due to lack of relevant data in the included studies.

We quantified the risk of developing CAP in current smokers to be similar to the association of smoking with asthma (RR 1.61; 95% CI, 1.07–2.42), idiopathic pulmonary fibrosis (OR 1.58; 95% CI 1.27–1.97), obstructive sleep apnoea (RR 1.97; 95% CI 1.02–3.82), stroke (HR 1.58; 95% CI 1.40–1.78) and acute coronary syndrome (HR 1.98; 95% CI 1.75–2.25) though lower than that of developing lung cancer (HR 13.1; 95% CI 9.90–17.3) and COPD (RR 4.01; 95% CI, 3.18–5.05).[35–38]

Our findings advance the descriptive presentation in two previous systematic reviews which reported that smoking is an independent modifiable risk factor for developing CAP alongside other lifestyle factors including alcohol abuse, low body mass index, having regular contact with children and poor dental hygiene.[10,39] Smoking also has an indirect effect on the risk of CAP as it is associated with COPD and poor dental health which are themselves independent risk factors for developing CAP. [24,28,35,40,41] This indirect effect has not been quantified.

In a cohort of immunocompetent adults aged 18–64 years old with invasive pneumococcal disease (IPD), Nuorti *et al*. showed that passive smoking was an independent risk factor (OR 2.5; 95% CI 1.2–5.1) with increased risk observed with longer duration of passive smoke exposure.[42] Although passive smoking was not associated with increased risk of CAP in adults of all ages, it is noteworthy that meta-analysis of two studies showed an increased risk in those ≥65 years; one study recruited patients from primary care (exposed to passive smoke at home) and the other following hospitalisation for CAP (exposed to any passive smoke). A combination of host factors such as comorbidities that accompany advanced age, polypharmacy and immune senescence as well as social factors including poor nutrition and crowding or long-term residential care may contribute to the increased risk of infection seen in the elderly. [43]

We were able to perform dose-response analyses using data from five studies. Our analyses confirmed that higher levels of smoking exposure are associated with higher risks of developing CAP. This is consistent with data from IPD where a linear dose-response relationship with number of cigarettes smoked daily has been reported.[44] In the two studies that combined current and ex-smokers in their analyses, the dose-response relationship between amount of smoking and risk of CAP was linear, whereas in the remaining three studies which had current smokers, the relationship was non-linear. An individual patient data analysis would be required to determine how the differences in the categorisation of smoking status influence the linearity of the dose-response relationship.

### Tobacco smoking and infection: Immune mechanisms

In addition to structural mechanisms mentioned in the ‘Introduction’ section,[6,7,45] smoking may increase the risk of systemic infections by causing changes in cellular and humoral immune system function.[46] Smoking impairs polymorphonuclear leukocyte function which plays a significant role in the host defence against bacterial infection (depressed neutrophil migration and leukocyte chemotaxis),[47,48] decreases CD4^+^ T cell counts which results in reduction of B cells that secrete antibodies (thus lowering serum immunoglobulin levels by approximately 10%),[49–53] increases CD8^+^ T cell counts,[54] and decreases secretion of pro-inflammatory cytokines such as IL-1 and IL-6.[55,56] Nicotine from tobacco smoking can also suppress natural killer (NK) cell activity; NK cells are usually activated as part of the early immune surveillance response against viral infections.[57]

### Effect of tobacco smoking cessation

The only study included in this review that reported time from smoking cessation to development of CAP was a population-based case-control study by Almirall *et al*. which reported that the risk of CAP reduced by 50% (OR) after five years of smoking cessation.[23] In a study of invasive pneumococcal disease (IPD), the risk of IPD in ex-smokers reduced by 14% annually and to that of never smokers about 13 years after smoking cessation.[44] These observations alongside the results from this meta-analysis support the notion that ex-smokers have a lower risk of CAP than current smokers and that this risk decreases with duration of smoking cessation.

Why ex-smokers remain at risk of CAP is unclear. Bacterial adherence is crucial in the pathogenesis of infection. Ex-smokers have been shown to have increased in vitro adherence of *Streptococcus pneumoniae* to buccal epithelial cells for up to three years after smoking cessation which may contribute to the increased risk of CAP.[45] In terms of alterations to immune function, reports have been mixed; one study found a significantly lower proportion of NK cells in ex-smokers who had stopped smoking for over 20 years compared to never smokers (mean duration since smoking cessation of 10.7 years)[58], whereas in another study of 10 ex-smokers where duration since smoking cessation ranged from six weeks to 10 years (mean = 4 years), NK cell activity was comparable to that of never smokers.[59] In heavy smokers (≥50 pack-years), smoking cessation for six weeks has been associated with a return of the CD4/ CD8 ratio to normal.[60], Therefore, although some of the immune related effects of smoking may be relatively rapidly reversed upon stopping smoking, other effects may be more prolonged, or possibly irreversible.

### Strengths and limitations

This review comprehensively summarises the current body of knowledge regarding the effect of tobacco smoking on the risk of developing CAP and was reported in accordance with PRISMA checklist (S2 File). Eligibility criteria were strictly applied to ensure identified studies only included patients with CAP, hence excluding hospital-acquired pneumonia, aspiration pneumonia, active pulmonary TB and post-obstructive pneumonia secondary to thoracic malignancy. Overall, included studies were of moderate quality and no language restrictions were applied. The statistical heterogeneity for the meta-analysis varied across the different analysis, ranging from low (<25%) to high (>75%) level and did not change significantly in the sensitivity analysis.

An important limitation was the various ways in which smoking status was defined and smoking ‘dose’ quantified. For instance, the lack of distinction of ‘ever’ and ‘not current’ smokers from ‘ex-smokers’ precluded seven studies from the meta-analysis involving ex-smokers. Definitions of CAP also varied, though to a lesser extent. Studies that did not adopt the ‘gold standard’ of radiologically confirmed CAP (n = 10 studies) may have captured some cases of LRTI or acute bronchitis instead. Non-pneumonic respiratory tract infections (RTIs) are generally considered to be more likely to be caused by viral pathogens instead of bacterial pathogens.[61] However, there are no comparative data to suggest a differential effect of smoking on the occurrence of viral versus bacterial RTIs. Therefore, inclusion of these studies is not expected to exert a major bias on pooled results.

### Implications

This review provides good evidence to support recommendations for smoking cessation as well as avoidance of passive exposure to tobacco smoke, particularly in persons at high risk of developing pneumonia. Patients who recover from an episode of CAP are recognised to be at risk of recurrent CAP[62–64]. Therefore hospitalisation with CAP provides a valuable ‘teachable moment’ when smoking cessation should be promoted. [65–67]

Further research is warranted to establish why and for how long ex-smokers continue to be at higher risk of developing CAP following smoking cessation, compared to those who have never smoked. For future studies, a more standardised approach to reporting pack-years of smoking instead of qualitative descriptions with variable definitions would facilitate comparisons and synthesis of data.

## Supporting information

S1 File Search strategies. (DOCX)

S2 File PRISMA checklist. (DOC)

S1 Table Characteristics of 27 included studies for systematic review; ordered by year, author. (DOCX)

S2 Table Risk of bias for included studies (using Newcastle Ottawa Scale). (DOCX)
