# Supplementary material for: Effect of tobacco smoking on the risk of developing community acquired pneumonia: A systematic review and meta-analysis
Source: PLoS One. 2019 Jul 18;14(7):e0220204. doi: 10.1371/journal.pone.0220204 (PMC6638981; doi:10.1371/journal.pone.0220204)
Supplement: S1 File — (DOCX) [file pone.0220204.s001.docx]

# S1 File: Search Strategies

## MEDLINE (Ovid)

1. exp smoking/ or smok*.mp.
2. exp smoking cessation/ or smoking cessation.mp.
3. exp “tobacco use”/ or exp Tobacco/ or exp tobacco products/ or exp “tobacco use disorder”/ of tobacco.mp.
4. exp tobacco smoke pollution/ or passive smok*.mp. or second?hand smok*.mp.
5. (nicotine* or cigar*).mp.
6. cotinine*.mp.
7. 1 or 2 or 3 or 4 or 5 or 6
8. exp Community-Acquired Infections/
9. exp pneumonia/ or pneumon.mp.
10. 8 OR 9
11. community acquired pneumonia.mp.
12. 10 OR 11
13. observational study.mp. or exp observational study/ or exp epidemiologic methods/
14. prospective study.mp. or exp prospective studies/
15. exp cohort studies/
16. case-control study.mp. or exp case-control studies/
17. 13 OR 14 OR 15 OR 16
18. 7 AND 12 AND 17
19. limit 18 to humans

## Embase (Ovid)

1. exp Smoking/ or smok*.mp.
2. exp Smoking Cessation/ or smoking cessation.mp.
3. exp “Tobacco Use”/ or exp Tobacco/ or exp “Tobacco Use Disorder”/ or tobacco.mp.
4. exp Tobacco Smoke Pollution/ or passive smok*.mp. or second?hand smok*.mp.
5. (nicotine* or cigar*).mp.
6. cotinine*.mp.
7. 1 or 2 or 3 or 4 or 5 or 6
8. exp Community-Acquired Infections/
9. exp pneumonia/ or pneumon*.mp.
10. 8 and 9
11. community acquired pneumonia.mp.
12. 10 or 11
13. observational study.mp. or exp Observational Study/ or exp Epidemiologic Methods/
14. prospective study.mp. or exp Prospective Studies/
15. exp Cohort Studies/
16. case-control study.mp. or exp Case-Control Studies
17. 13 or 14 or 15 or 16
18. 7 and 12 and 17
19. limit 18 to humans

## PsycINFO (Ovid)

1. exp SMOKING CESSATION/ or smoking.mp. or exp PASSIVE SMOKING/ or exp TOBACCO SMOKING/
2. tobacco.mp.
3. second?hand smoke.mp.
4. exp NICOTINE/ or nicotine.mp.
5. cigar.mp.
6. cotinine.mp.
7. 1 or 2 or 3 or 4 or 5 or 6
8. community acquired infections.mp.
9. pneumonia.mp. or exp PNEUMONIA/
10. community acquired pneumonia.mp.
11. 8 or 9 or 10
12. exp observation methods/ or observational stud*.mp.
13. epidemiolog*.mp. or exp EPIDEMIOLOGY/
14. epidemiologic methods.mp.
15. exp Prospective Studies/ or prospective stud*.mp.
16. cohort stud*.mp.
17. case-control study.mp.
18. 12 or 13 or 14 or 15 or 16 or 17
19. 7 and 11 and 18

## Web of Science

1. smoking
2. smoking cessation
3. tobacco
4. tobacco products
5. tobacco cessation
6. passive smoking
7. second hand smoke
8. nicotine
9. cigar
10. cotinine
11. 1 OR 2 OR 3 OR 4 OR 5 OR 6 OR 7 OR 8 OR 9 OR 10
12. observational study
13. epidemiology
14. prospective study
15. cohort study
16. case control study
17. 12 OR 13 OR 14 OR 15 OR 16
18. community acquired infection
19. pneumonia
20. community acquired pneumonia
21. 18 AND 19
22. 20 OR 21
23. 11 AND 17 AND 22
