# Supplementary material for: Effect of tobacco smoking on the risk of developing community acquired pneumonia: A systematic review and meta-analysis
Source: PLoS One. 2019 Jul 18;14(7):e0220204. doi: 10.1371/journal.pone.0220204 (PMC6638981; doi:10.1371/journal.pone.0220204)
Supplement: S1 Table — (DOCX) [file pone.0220204.s003.docx]

**SUPPORTING INFORMATION**

Effect of tobacco smoking on the risk of developing community acquired pneumonia:

A systematic review and meta-analysis

Vadsala Baskaran^1^, Rachael L Murray^2^, Abby Hunter^2^, Wei Shen Lim^1^, Tricia M McKeever^2^

^1^ Department of Respiratory Medicine, Nottingham University Hospitals NHS Trust, Nottingham, United Kingdom

^2^ Division of Epidemiology and Public Health, University of Nottingham, Nottingham, United Kingdom

# S1 Table: Characteristics of 27 included studies for systematic review; ordered by year, author.

| **First Author** | **Year** | **Study design** | **Country** | **Setting** | **Study population** | **Final study number** | **How is smoking status measured?** | **Smoking categories*** | **CAP definition** |
| --- | --- | --- | --- | --- | --- | --- | --- | --- | --- |
| **Case –control studies** | | | | | | | | | |
| Almirall | 1999 | Multicentre prospective | Spain | Mixed | Mean age: men 56.1 (SD 19), women 51.1 (SD 20.7)  Excluded a secondary concurrent disease or a noninfectious origin, HIV, active cancers, dementia. | Cases 205/ Controls 475 | Questionnaire | Never Ever  Ex Current | Acute lower respiratory tract infections for which antibiotics were prescribed, associated or not with new focal signs on examination of the chest and radiographic infiltrate indicative of pneumonia. |
| Almirall | 1999 | Multicentre retrospective | Spain | Mixed | Mean age: men 56.1 (SD 19), women 51.1 (SD 20.7) Male: 54.6%  Excluded HIV, active cancer, aspiration, active lung TB, nursing homes residents, HAP, dementia, non-contactable. | Cases 205/ Controls 475 | Questionnaire completed by nurses/ doctors | Never Ex Current | Acute lower respiratory tract infections for which antibiotics were prescribed, associated or not with new focal signs on examination of the chest and radiographic infiltrate indicative of pneumonia |
| Farr | 2000 | Multicentre prospective | UK | Primary care | Mean age: cases 54, controls 44 Male: cases 55%, controls 45% | Cases 66, Controls 489 | Questionnaire | Never ‡  Ex  Current Passive | Acute lower respiratory tract infection for which an antibiotic was prescribed associated with new focal signs on chest examination and new radiographic pulmonary shadowing. |
| Farr | 2000 | Multicentre prospective | England | Hospital | Age: cases 44.9, controls 26.5% ≥60  Male: cases 60.7%, controls 54.3%  Excluded if CAP was not the main reason for admission, expected terminal event, distal to a bronchial event, pulmonary TB, control had CAP as an adult. | Cases 178, Controls 385 | Questionnaire | Never  Current (mild, heavy)  Passive | Acute respiratory illness with radiological pulmonary shadowing which was at least segmental or present in more than one lobe.Two control subjects for each case were selected at random from the electoral registers for the main catchment areas of the hospitals where the cases were treated. |
| Piednoir | 2003 | Single centre retrospective | France | Hospital | Mean age: 88.3 (SD 4.1) Male: 68%  Excluded if not confirmed radiologically. | Cases 101, Controls 101 | Medical records | Not current  Current | Radiological diagnosis of one or more opacities + one of the below: bacterial infection, temp > 38, legionella, cough, wheeze, DIB |
| § Greig | 2004 | Single centre retrospective | Australia | Community | Median age: 64 Male: 57%  Included participants who visited an aquarium.  Controls excluded if had clinical disgnosis of CAP | Cases 104, Controls 201 | Telephone interview using standardised questionnaire | Never  Current | Fever, cough, pneumonia in the 2/52 after a visit to Melbourne Aquarium/ close vicinity + confirmed by ≥ one test: (1) +ve urinary antigen test (2) ≥ 4x (to ≥ 128) rise in antibody titre against L pneumophila between paired acute and convalescent phased sera (3) Isolation of Legionella spp. from resp secretions (4) stable high (>512) titre in convalescent serum |
| Bai | 2007 | Single centre retrospective | China | Hospital | Age: cases 76.90, controls 74.64 Male: 75% | Cases 128, Controls 306 | Questionnaire completed by patients | Current Ever | Not reported |
| Almirall | 2008 | Multicentre prospective | Spain | Primary care | Cases: mean age: 54.6 (SD 20.7) (women) – 58.6 (SD 19.8) (men)  Male: 52.9%  Controls: Mean age : Controls 54.6 (SD 20.6) (women)– 58.9 (SD 19.6) (men) Male: 52.6%  Excluded aspiration, active pulmonary TB, HAP, nursing homes residents. | Cases 1336/ Controls 1326 | Questionnaire | Never  Ex  Current Passive | Acute lower respiratory tract infection for which antibiotics had been prescribed, with the appearance of previously unrecorded focal signs on physical examination of the chest and new radiological findings suggestive of pneumonia infiltrate. |
| Jackson | 2008 | Multicentre  retrospective | USA | Mixed | Age: 65-94 Male: 51%  Excluded nursing home/ hospice care residents, if they had <2 visits to Group Health providers in previous 2 years. | Cases 1173, Controls 2346 | Medical records | Never Ever | ICD-9 codes for CAP; These episodes were then validated either by review of electronically available reports of chest radiographs obtained within 30 days before or after the visit or, for events in the hospital, by review of the hospital records. |
| Tas | 2008 | Single centre retrospective | Turkey | Hospital | Mean age: cases 22.18 (SD 1.22), controls 22.18 (SD 1.23) Male: 100%  Excluded ex-smokers, soldiers with chronic diseases | Cases 58, Controls 580 | Interview | Never Current | Patients with clinical, laboratory and radiological findings compatible with pneumonia were hospitalized |
| Loeb | 2009 | Multicentre retrospective | Canada | Hospital | Mean age: cases 79.1 (SD 7.6),controls 74.4 (SD 6.7)  Male: cases 60.4%, controls 31.5%  Excluded nursing home residents, infection at another site (in addition to CAP) at the time of enrolment, and residence outside the study catchment areas. Control participants were excluded if they had been diagnosed with CAP in the previous 12 months or had any other active infection. | Cases 717, Controls 867 | Questionnaire completed by interviewer | Ever  Passive | ≥ 2 of the following symptoms and signs: temperature higher than 38.1C, productive cough, chest pain, shortness of breath, or crackles on auscultation., new opacity on a chest radiograph interpreted by a radiologist as being compatible with pneumonia. |
| Gau | 2010 | Single centre retrospective | USA | Hospital | Mean age: cases 80.3 (SD 8.5)/, controls 79.8 (8.1) Male: cases 39%, controls 33%  Excluded aspiration, active lung cancer/ metastatic disease, ventilator-associated/ HAP, death as inpatient, haemodialysis patients, incomplete records, patients who could not recall medication use | Cases 194, Controls 952 | Medical records/ standardised form | Never ‡  Ex Current | Discharge diagnosis and further confirmed by the report of the radiographic findings (a new infiltrate or consolidation) suggestive of pneumonia. |
| Teepe | 2010 | Multicentre prospective | Dutch | Primary care | Age: cases 34.3, controls 32.1 Male: cases 39.1%, controls 35.5% | Cases 156, Controls 468 | Questionnaire completed by trained research nurse at interview | Not current  Current | International Classification of Primary Care (ICPC) code R81:Either a confirmation by radiographyor the presence of ≥ 3 of the following signs/ symptoms:   - decreased intensity of breath sounds; - dullnesson chest percussion; - inspiratory crackles; - increased vocal resonance; - fever - local chest pain on deep inhalation |
| Almirall | 2014 | Multicentre prospective | Spain | Primary care | Median age: cases 65 (14-96), controls 63 (15-100) Male: cases 25%, controls 29%  Excluded aspiration, HAP, active lung TB, if another non-infectious respiratory disease was later confirmed. | Cases 471/ Controls 532 | Questionnaire completed by trained physician/ nurse at interview | Never; passive  Never; non- passive | Acute lower respiratory tract infection for which antibiotics had been prescribed with appearance of new or previously unknown focal signs on physical examination and radiography of the chest |
| **Cohort studies** | | | | | | | | | |
| § Conley | 1996 | Multicentre prospective & retrospective | USA | Not specified | Median age: 36-38 Male: 100%  Included HIV infected men  Excluded ex-smokers and intermittent smokers. | 232 | Questionnaire | Never Current | Illness must be severe enough to require a visit to a physician, a diagnosis of ‘pneumonia’ by the clinician and his prescription of an antibiotic. |
| Baik | 2000 | Multicentre prospective | USA | Community | Age: men 44-79, women 27 -44  Excluded HAP, diagnosis of pneumonia not identified by medical records or by supplemental questionnaire, CAP before the beginning of the study, those who didn’t respond to questions on body weight and physical activity, women with probable or possible CAP. | 104491 | Questionnaire | Never Ex  Current | First documented physician diagnosed CAP  Men: CXR infiltrate documented in the report Women: self-report |
| Jackson | 2004 | Multicentre  prospective | USA | Mixed | Age: 53% 65- 74, 38% 75 - 84 Male: 42%  Excluded HAP | Hospitalised 1266 OPD 1881 | Outpatient visit documentation for GHC | Not current Current | For CAP that required hospitalisation: Treating physician considered CAP as the aetiology  For outpatient CAP: ICD 9 codes, chart review indicated that pneumonia was the most likely diagnosis, CXR within 14 days of visit, & if the patient had not been hospitalized in the prior 7 days. |
| O-Meara | 2005 | Multicentre prospective | USA | Community | Mean age: Hospitalised 75, Not hospitalised 72.6 Male: hospitalised 49%, not hospitalised 42%  Excluded institutionalised, not ambulatory at home, under hospice care, receiving radiation or chemotherapy for cancer, not expected to remain in the area for ≥ 3 years, unable to be interviewed | 5888 | Interview | Never ‡  Current Ever | Pneumonia identified by codes assigned to hospital discharge diagnoses according to ICD-9 codes (481, 482, 486) |
| § Gordin | 2008 | Multicentre prospective | 33 countries | Not specified | Median age: 43 Male: 72.8%  Included HIV infected persons | 5472 | Standardised case report by trained interviewer | Never Ex Current | (1) ‘‘confirmed’’: compatible clinical and radiographic evidence with histologic or microbiologic support  (2) ‘‘probable’’: signs and symptoms of pneumonia with compatible radiographic abnormalities |
| § Mannino | 2009 | Multicentre prospective | USA | Community | Age ≥ 45 Male: 44.5% | 214 | Questionnaire | Never Ex Current | Hospitalisations that include pneumonia discharge code (ICD-9 codes 480 - 486) within 36 months |
| § Chauny | 2012 | Multicentre prospective | Canada | Hospital | Mean age: 53 (SD 17) Male: 63%  Included patients with minor thoracic injury  Excluded spontaneous rib fracture not associated with trauma, confirmed diagnosis of hemothorax, pneumothorax, lung contusion, or any other significant internal thoracic or abdominal injury at their initial ED visit, if they could not be followed as outpatients, or if the interval from time of injury to ED visit exceeded 3 days. | 1057 | Questionnaire completed by patients | Never Ex Current | Presence of pneumonia on radiologic reports, with compatible patient complaints and physical examination in the first 2 weeks, or the reported diagnosis of delayed pneumonia, with antibiotic administration. |
| Takahashi | 2013 | Single centre prospective | Vietnam | Hospital | Median age: 50 Male: 52%  Excluded HAP, no interpretable CXR. | 174 | Standardised form | Never  Ever | Hospitalised with ≥ 2 of the following: (1) fever and/or cough  (2) fast and/or difficulty of breathing (3) any additional severe symptoms including respiratory rate over 30 per minute, SpO2 under 90%, systolic blood pressure under 90 mmHg, pulse rate more than 130/min, white blood cell count over 20,000 or under 4,000 cells/μL, CRP over 20 mg/dl, dehydrated, altered consciousness and other worse general status.  A senior pulmonologist and two physicians interpreted all chest X-ray films independently. A case was categorised as CAP if ≥ 2 evaluators agreed on the presence of consolidation. |
| Yende | 2013 | Multicentre prospective | USA | Community | Mean age: 59.23 (SD 10.06) Male: 44.1%  Excluded:  CHS: wheelchair bound, unable to participate in the examination at the field centre, definitive plans to leave the area, active cancer.  Health ABC: recent treatment of cancer, severe dementia, plans of leaving the area within 3 years. | 16260 | Self-report | Never  Ex  Current | ICD 9 code 480 - 487 |
| § Attia | 2015 | Multicentre  prospective | USA | Hospital | Median age: 44-50  Male: 98%  Included HIV infected veterans | 41993 | Electronic health record/ Veterans Affair database | Never  Ex  Current | Inpatient ICD-9 code for CAP |
| Breitling | 2016 | Multicentre prospective | Germany | Primary care | Age: 50-75 Male: 44.6% | 9419 | Questionnaire | Never Ex Current | First episode of pneumonia reported either by the participants or their physicians |
| Jackson | 2016 | Multicentre  prospective | USA | Community | Age ≥65 Male: 41% | 3375 | Questionnaire completed by interviewer | Never Ex Current | (ICD-9-CM) codes 480–487.0 or 507.0 assigned to outpatient and inpatient medical encounters.  • Presumptive pneumonia episodes were considered validated if manual review of chest radiograph reports within 30 days of first pneumonia diagnosis indicated the presence of an infiltrate not known to be chronic. • If no CXR, validated by reviewing hospital admission, consultation, and discharge summaries. |
| § Braeken | 2017 | Multicentre retrospective | UK | Primary care | Mean age: 67 Male: 55.2%  Included patients with COPD  Excluded asthma, pneumonia in preceding 3 months, pulmonary TB and unknown smoking status. | 254 275 | CPRD database | Never Ex Current | Physician-recorded pneumonia diagnosis, identified by read codes. |

**Abbreviations**

SD: standard deviation, HIV: human immunodeficiency virus, TB: tuberculosis, HAP: hospital acquired pneumonia, ED: emergency department, ICD: International Classification of Diseases, COPD: chronic obstructive lung disease, CHS: Cardiovascular Health Study, Health ABC: Health, Aging, and Body Composition

**Legend**

* Smoking categories have been renamed from original studies in this table to match Table 1 for consistency: never and non-smokers in the ‘never’ category; lifetime smoking history of > 100 cigarettes, ‘current/ past’ and ‘current/ex-‘ in the ‘ever’ category, ex-, former and past smokers in the ‘ex’ category; and current and active smokers in the ‘current’ category.

‡Studies which had information for ‘never’ smoking category although did not explicitly state the category.

§ Studies which had selected clinical population
