# Supplementary material for: Effect of tobacco smoking on the risk of developing community acquired pneumonia: A systematic review and meta-analysis
Source: PLoS One. 2019 Jul 18;14(7):e0220204. doi: 10.1371/journal.pone.0220204 (PMC6638981; doi:10.1371/journal.pone.0220204)
Supplement: S2 Table — (DOCX) [file pone.0220204.s004.docx]

# S2 Table: Risk of bias for included studies (using Newcastle Ottawa Scale)

| First Author | Year | Study quality | | | |
| --- | --- | --- | --- | --- | --- |
|  |  | Selection  (max= 4) | Comparability  (max= 2) | Outcome  (max= 3) | Total |
| Almirall | 1999 | 4 | 2 | 1 | 7 |
| Almirall | 1999 | 4 | 2 | 1 | 7 |
| Almirall | 2008 | 4 | 2 | 1 | 7 |
| Almirall | 2014 | 4 | 1 | 1 | 6 |
| Attia | 2015 | 2 | 2 | 2 | 6 |
| Bai | 2007 | 2 | 2 | 0 | 4 |
| Baik (men) | 2000 | 2 | 2 | 3 | 7 |
| Baik (women) | 2000 | 2 | 2 | 2 | 6 |
| Braeken | 2017 | 4 | 2 | 2 | 8 |
| Breitling | 2016 | 2 | 2 | 2 | 6 |
| Chauny | 2012 | 1 | 0 | 1 | 2 |
| Conley | 1996 | 2 | 2 | 2 | 6 |
| Farr | 2000 | 4 | 2 | 2 | 8 |
| Farr | 2000 | 4 | 2 | 1 | 7 |
| Gau | 2010 | 3 | 2 | 2 | 7 |
| Gordin | 2008 | 2 | 2 | 1 | 5 |
| Greig | 2004 | 3 | 2 | 1 | 6 |
| Jackson | 2004 | 2 | 2 | 2 | 6 |
| Jackson | 2008 | 3 | 1 | 2 | 6 |
| Jackson | 2016 | 3 | 2 | 2 | 7 |
| Loeb | 2009 | 4 | 2 | 1 | 7 |
| Mannino | 2009 | 1 | 2 | 3 | 6 |
| O-Meara | 2005 | 2 | 2 | 2 | 6 |
| Piednoir | 2003 | 2 | 2 | 1 | 5 |
| Takahashi | 2013 | 1 | 2 | 1 | 4 |
| Tas | 2008 | 4 | 1 | 1 | 6 |
| Teepe | 2010 | 3 | 2 | 1 | 6 |
| Yende | 2013 | 2 | 2 | 2 | 6 |
